# The Rival Note Takers

**Published:** 1830-01-01

**Authors:** 


### THE RIVAL NOTE TAKERS.

Intereunt omnes.

Mr. Lawrence has revoked his permission to some six or eight journals to publish his lectures every Saturday morning. Poor gentleman! We dare say he must have felt some such qualms as David is said to do at Sternold and Hopkins' translation of his Psalms, when he witnessed his unfortunate sentences twisting and writhing into all their fantastic forms under the combined exertions of short-hand writer and printer's devil. Poor gentleman! He might have said to the public, like the frog in Æsop, this is sport to you but death to me! The hebdomadals made holiday indeed, fattening and glutting on the Lecturer with a lively presentiment no doubt of the short lived nature of their gorge. Mr. Lawrence was served up regularly in yellow and in green and in Heaven knows how many colours besides, till at length he became like a sloughing fungus surrounded by a halo of "inflammation." And then the quarrels of the respective cooks; why you would have thought they were so many dogs over a bone,

> Gorging and growling o'er carcass and limb,
> They were too busy to look at him.

In one of those Christmas pantomimes with which when youngsters we used to be delighted, and which (shall we own the "soft impeachment?") still occasionally tickle our more sober fancies, in one of those pleasant amusements we say, a too attractive beau is placed amongst a party of ladies of somewhat strong passions and easy virtue. Each in her eagerness to monopolize the dear object seizes hold of an arm, a finger, or a toe, a violent struggle amongst the fair candidates ensues, and at length the gentleman is pulled, like poor Orpheus in Thrace, into a thousand pieces. Such a method of shewing one's affection though agreable no doubt to the looker on, is naturally rather unpleasant to the party particularly interested. Now Mr. L. was placed in much the same situation as the beau in the pantomime. Each of the journals which blazoned his fame and reported his lectures was naturally jealous lest its rival should receive an ogle, a squeeze, or an amorous sigh from the common object of either's devotion. One of them, whether unusually fond or unguardedly bold, ventured on the common *ruse* of love, and protested or rather whispered that it had been made happy by favours secret, sweet, and precious. A billet-doux was hinted to have been received from Whitehall Place; and Fame, that tattling jade, now stalking as a burly Churchwarden of St. Giles's, now skulking in the sooty form of that "devil which printers own," spread the loving lie in our modern Babylon. The opposite party at the *bruit* of this horrid rumour sank at first into a sort of stupor, accompanied with such low muttering delirium, that many of the friends began to entertain very serious apprehensions of fatal oppression on the brain. These apprehensions fortunately proved to be unfounded, for after a *green* and yellow melancholy which lasted about a week, jealousy and distraction again took possession of the mind, and the never-to-be-sufficiently appeased individual hurried to the fountain head to ascertain whether or not the hateful report was true. To the unutterable joy of the applicant it was found to be totally false, but the objurgations, the tearing of hair, the shocking names, and hard blows that ensued between the rivals it was really dreadful to hear or see. The lecturer appeared to be sadly bothered with his doxies, and sighed, for sing he could not, the well-known Macheathian stanza on a similar occasion, with wonderful pathos and effect.

One of the parties in particular, arrayed in a loose jalap-coloured wrapper, which the poor soul was always observed to be particularly fond of decorating with figures of hats and bonnets and babies caps, she, we regret to say, became so outrageous that even her best friends put their hands to their ears. A singular hallucination appeared to be present to the demented creature's sensorium. She thought she was contemplating an old ivy mantled tower, with various birds of night, and particularly bats, flitting in the gloom and nesting in the ruins. With her eye "in a fine phrenzy rolling" she invoked all these repulsive animals with great ardour and excitement, screaming out in a remarkably harsh and vulgar tone, bats—vampires—blood-suckers—corruptionists—toads—butchery—murder—havoc—tyranny—non-medical coroners—select vestries—Lincoln's Inn—St. Giles's—infamy—curses—damn——n—and the like, till the blood of the byestanders positively curdled in their veins. Many attempts were made to pacify or shame her but all to no purpose, for the monomaniacal orgasm was above control. It was singular that when at the very height of her fury, she imagined herself a great blessing sent down from above to purify and cleanse the old building in question, although it was quite evident to those who approached her at all closely that the peculiarly disagreeable maniacal odour was very strong upon her. Her habits of cleanliness too were so completely forgotten, that her stench was like the rod of Aaron, and completely overpowered and absorbed the effluvia of the whole of the other animals.

With regard to the lecturer, she was observed to entertain some very curious ideas respecting him. At one time she considered him a demigod, and stroked his beard with such fondness and regard that it actually drew tears of sensibility from the eyes of those who witnessed her. But he having found it expedient to go into a chamber of the building, where several elderly looking persons were seated at a table questioning some very sheepish looking young men, and having with much difficulty got a chair amongst them, the affections of the poor soul were evidently weaned from him. She took it much to heart, and appeared to upbraid him privately, but was deterred from accusing him publicly of infidelity partly from the dread of many friends who surrounded him, and still more from her anxiety to extract something from his pocket. What this was could never be exactly ascertained, one set of persons declaring that it was a note to prevent an execution, others that it was a large bundle of papers, tied with red tape, and appearing to be his lectures. Whatever it was, the refusal to surrender it threw her into such an accession of the most furious of furies that well founded fears were entertained of her putting a violent end to herself, her rival, the neighbours, or even the once dear lecturer. Fortunately at this crisis a strong party of the New Police happened to come by on their way to the St. Giles's district, and they judged it expedient on all accounts to lodge her in that watch-house for the night. We regret to say that we have had no means of ascertaining the subsequent fate of the unfortunate creature.

---

